# SARS-CoV-2 Infection in Asymptomatic Patients Hospitalized for Cardiac Emergencies: Implications for Patient Management

**DOI:** 10.3389/fcvm.2020.599299

**Published:** 2020-12-18

**Authors:** Thorsten Kessler, Jens Wiebe, Tobias Graf, Heribert Schunkert, Adnan Kastrati, Hendrik B. Sager

**Affiliations:** ^1^Deutsches Herzzentrum München, Klinik für Herz- und Kreislauferkrankungen, Technische Universität München, Deutsches Zentrum für Herz-Kreislauf-Forschung (DZHK) e.V., Partner Site Munich Heart Alliance, Munich, Germany; ^2^Universitätsklinikum Schleswig-Holstein, Medizinische Klinik II, Deutsches Zentrum für Herz-Kreislauf-Forschung (DZHK) e.V., Partner Site Hamburg/Kiel/Lübeck, Lübeck, Germany

**Keywords:** cardiac emergencies, SARS-CoV-2, COVID-19, personal protective equipment (PPE), screening

## Abstract

**Background:** The coronavirus disease (COVID-19) pandemic imposed diverse challenges on the health care system. Morbidity and mortality of non-COVID-19 emergencies might also have changed because hospitals may not be able to provide optimal care due to restructured resources and uncertainties how to deal with potentially infected patients. It has been recommended to stratify treatment of cardiovascular emergencies according to cardiovascular risk. However, data on the prevalence of asymptomatic SARS-CoV-2 infection in patients presenting with cardiac emergencies remain scarce.

**Methods:** We retrospectively analyzed patients' data from a tertiary cardiology department between April 15 and May 31, 2020. All patients were screened on admission for COVID-19 symptoms using a questionnaire and body temperature measurements. All hospitalized patients were routinely screened using nasopharyngeal swab testing.

**Results:** In total, we counted 710 urgent and emergency admissions. Nasopharyngeal swab tests were available in 689 (97%) patients, 409 and 280 of which presented as urgent and emergency admissions, respectively. Among 280 emergency admissions, none tested positive for SARS-CoV-2.

**Conclusion:** In cardiac emergency patients which were screened negative for COVID-19 symptoms, the prevalence of SARS-CoV-2 infection in regions with a modest overall prevalence is low. This finding might be helpful to better determine timing of emergency procedures and reasonable usage of protective equipment during the COVID-19 crisis and the future.

## Introduction

The coronavirus disease (COVID-19) pandemic imposed diverse challenges on health care providers and hospitals. For instance, hospitals needed to rapidly redistribute and reorganize resources to treat acutely ill COVID-19 patients while keeping up with other emergencies. At the same time outmost attention had to be spent on containing severe acute respiratory syndrome coronavirus 2 (SARS-CoV-2) to protect other patients and staff. Apart from rising numbers of COVID-19 patients, a change in the presentation pattern of non-COVID emergencies was observed. In that light, it was recently shown that hospital admissions for acute coronary syndrome (ACS) declined during the pandemic (1–4). Apart from a decrease in presentations, morbidity and mortality of non-COVID-19 emergencies might have changed because hospitals may not be able to provide optimal care due to restructured resources and uncertainties how to deal with potentially infected patients (5). Position papers consequently recommended to stratify treatment of cardiovascular emergencies according to cardiovascular risk: (1) only high-risk emergencies (e.g., ST elevation myocardial infarction) should be treated immediately with usage of personal protective equipment as in confirmed COVID-19 cases; (2) other emergencies and elective procedures should only be carried out after receiving results of SARS-CoV-2 testing (6–8). A better understanding of the prevalence of asymptomatic SARS-CoV-2 infections may help to guide timely management and reasonable usage of personal protective equipment without affecting the safety of staff and other patients.

We here sought to investigate the prevalence of SARS-CoV-2 infections in asymptomatic patients presenting with cardiac emergencies.

## Methods

### Study Cohort

The study protocol was approved by the institutional ethics committee (323/20 S) and conforms to the ethical guidelines of the 1975 Declaration of Helsinki. We retrospectively analyzed patients' data from a tertiary cardiology department which provides 24/7 interventional cardiac care between April 15 and May 31, 2020 (i.e., at the peak of the pandemic's first wave in the region).

### Screening of Patients

All patients were screened on admission for COVID-19 symptoms using a questionnaire and had their body temperature measured (ear thermometer). Patients were assigned as COVID-19 asymptomatic when none of the following criteria were met: body temperature ≥38.1°C, coughing, shortness of breath, runny nose, sore throat, or body aches. Additionally, patients were asked whether they had been in contact to a confirmed COVID-19 case or a patient suffering from fever and coughing without proven SARS-CoV-2 infection. Patients reporting shortness of breath were also regarded as COVID-19 asymptomatic if they did not report one of the other criteria. COVID-19 asymptomatic patients were required to wear standard surgical masks (no N95 or FFP2-3) throughout the entire stay. Hospital staff was also required to wear standard surgical masks at all times. These protective measurements were recently shown to reduce in particular the risk of SARS-CoV-2 infection for healthcare workers (9). N95 masks or FFP2-3 masks and further dedicated protective equipment were only used when treating SARS-CoV-2 confirmed or suspected patients.

### SARS-CoV-2 Testing

All hospitalized patients were routinely screened for SARS-CoV-2 using nasopharyngeal swab testing (SARS-CoV-2 real-time polymerase chain reaction assay, Mikrogen Diagnostik, Neuried, Germany) since April 15, 2020. Patients without COVID-19 symptoms were only planned to be tested at admission. Repeated testing was performed if patients developed symptoms or if a more recent test result was required for transferal to other treatment facilities.

## Results

**Figure 1:**
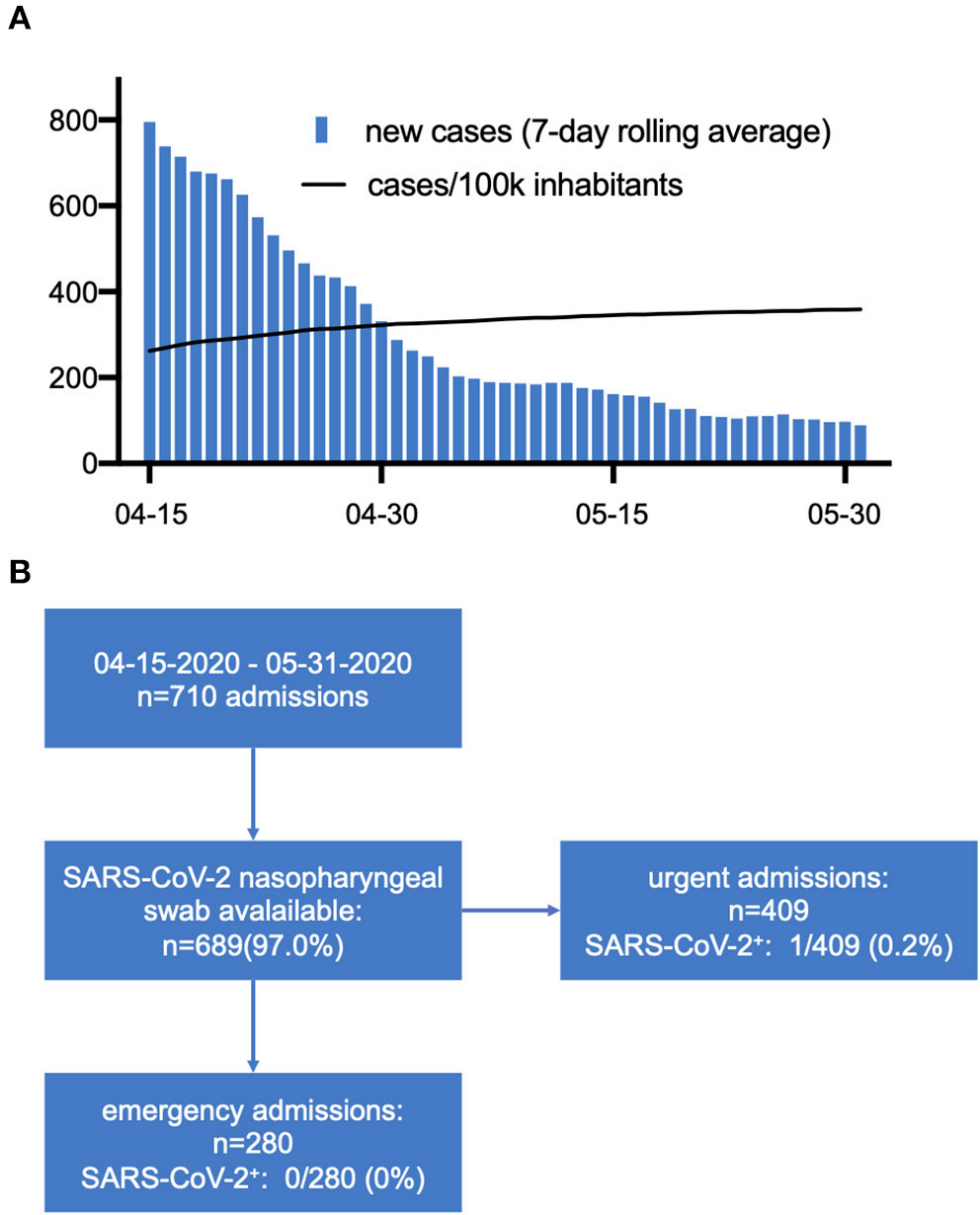
**(A)** Daily reported cases (Bayerisches Landesamt für Gesundheit und Lebensmittelsicherheit, accessed on 07-15-2020) and prevalence (Daily Situation Report of the Robert Koch Institute, accessed on 07-15-2020) of SARS-CoV-2 infections in the Free State of Bavaria during the study period. **(B)** Prevalence of asymptomatic SARS-CoV-2 infection.

Until May 31, 2020, a total of 710 patients presented and were included this analysis. Figure 1A displays the number of daily infections in the Free State of Bavaria, Germany and the prevalence of SARS-CoV-2 infections per 100,000 inhabitants during the study period. Nasopharyngeal swab tests were available in 689 (97%) patients, 409 and 280 of which presented as urgent [reasons: coronary 116/409 (28.4%), structural 42/409 (10.3%), heart failure 8/409 (2%), electrophysiology 209/409 (51.1%), other 34/409 (8.3%)] and emergency admissions, respectively. As a suspected SARS-CoV-2 infection may have reduced the likelihood of presenting with a non-emergency leading to an underestimation of the actual prevalence, we focused on the 280 patients admitted as cardiac emergencies. Baseline characteristics and reasons for admission are displayed in Table 1. None of these COVID-19 asymptomatic patients tested positive for SARS-CoV-2. During the hospital stay, 27 (9.6%) of patients were repeatedly tested with no test revealing a positive result.

**Table 1:** Baseline characteristics and reasons of admission for the patients presenting with cardiac emergencies during the study period.

	**Emergency admissions**
	***n* = 280**
Age, years ±*SD*	68.5 ± 15.0
Female gender, *n* (%)	103 (36.8)
**Comorbidities**	
COPD, *n* (%)	19 (6.8)
Diabetes, *n* (%)	60 (21.4)
Hypertension, *n* (%)	189 (67.5)
Coronary artery disease, *n* (%)	131 (46.8)
Peripheral artery disease, *n* (%)	26 (9.3)
Cerebrovascular disease, *n* (%)	34 (12.1)
Cancer, *n* (%)	30 (10.7)
Chronic renal dysfunction, *n* (%)	50 (17.9)
Immunodeficiency, *n* (%)	8 (2.9)
**Reasons for admission**	
Coronary, *n* (%)	97 (34.6)
Heart failure, *n* (%)	23 (8.2)
Structural, *n* (%)	7 (2.5)
Electrophysiology, *n* (%)	93 (33.2)
Other, *n* (%)	60 (21.4)

Coronary includes (suspected) acute coronary syndromes. Electrophysiology includes, e.g., tachycardia and bradycardia. Other includes, e.g., syncope, pulmonary embolism.

*COPD, chronic obstructive pulmonary disease; SD, standard deviation*.

In the total cohort, only one patient was diagnosed to be SARS-CoV-2 positive (Figure 1B). The patient was sent in home quarantine and treatment was scheduled to be performed after 14 days of quarantine and two subsequent negative nasopharyngeal swabs. This patient remained asymptomatic and no further testing was performed during quarantine.

## Discussion

This result needs to be reviewed in the context of the overall SARS-CoV-2 prevalence in the respective region during the observation period. During the study period, ~300 cases per 100,000 citizens were reported in the Free State of Bavaria. Thus, our data indicate that in cardiac emergency patients which were screened negative for COVID-19 symptoms, the prevalence of SARS-CoV-2 infection in regions with a modest overall prevalence is low. Under these circumstances, our findings indicate that a delay/deferral of emergency procedures due to waiting for SARS-CoV-2 test results may not be justified in emergency patients which are screened asymptomatic for COVID-19, but have an unclear SARS-CoV-2 infectious status. While our finding is in line with a recent report from Iceland, where in a random-sample screening of the population, 0.6% tested positive for SARS-CoV-2 (10), a study screening pregnant women admitted for delivery in New York City found that 13.5% of tested women were asymptomatic but tested positive (11).

In summary, the frequency of asymptomatic SARS-CoV-2 carriers among cardiac emergency patients is low when the overall prevalence of COVID-19 is modest. Consequently, emergency but also elective procedures may safely be carried out without delay and waiting for SARS-CoV-2 test results. Importantly, the safety of personnel and patients may be further increased by implementation of rapid or point-of-care tests which despite potential drawbacks [for an overview, see (12)] recently revealed promising results (13).

Our study was performed during a time period in which the prevalence of COVID-19 in Bavaria was rather low and our findings are therefore inherently not applicable in regions with higher prevalence. It was also previously shown that the highest sensitivity of SARS-CoV-2 was reached bronchoalveolar lavage fluid (14) and we may have missed SARS-CoV-2 infection due to only performing nasopharyngeal swab testing. Additional major limitations are the retrospective nature of this analysis and that the data are derived from a single center. The low prevalence of SARS-CoV-2 infection may therefore be due to chance and requires validation in further cohorts to draw definitive conclusions. Our data may nevertheless be helpful to better determine timing of emergency procedures and reasonable usage of protective equipment during the COVID-19 crisis and the future.

## Data Availability Statement

The original contributions presented in the study are included in the article/supplementary materials, further inquiries can be directed to the corresponding author/s.

## Ethics Statement

The studies involving human participants were reviewed and approved by Technische Universität München. Written informed consent for participation was not required for this study in accordance with the national legislation and the institutional requirements.

## Author Contributions

AK and HBS designed the study. TK, JW, TG, and HS contributed data. TK, AK, and HBS drafted the manuscript. All authors were involved in critically revising the manuscript.

## Conflict of Interest

The authors declare that the research was conducted in the absence of any commercial or financial relationships that could be construed as a potential conflict of interest.
